# Bilateral Testicular Loss due to Dog Bite in a Child

**DOI:** 10.21699/ajcr.v8i3.575

**Published:** 2017-05-01

**Authors:** Taketoshi Nara, Eiji Hisamatsu, Akiko Haruna, Yoshifumi Sugita

**Affiliations:** 1 Department of Urology, Akita University School of Medicine, Akita, Japan; 2 Department of Urology, Aichi Children’s Health and Medical Center, Obu, Japan; 3 Department of Urology, Kobe Children’s Hospital, Kobe, Japan

**Keywords:** Dog bite, Testicular loss, Genital trauma

## Abstract

Although animal bites are common, genital trauma caused by animal bites is rare. Here we report a case of bilateral testicular loss in an infant due to a dog bite. The patient was bitten by a friend’s dog while his mother was intoxicated and, therefore, did not receive immediate medical attention. Although initial treatment with subsequent genital reconstruction and hormone replacement is important for children with genital trauma, it is too important to make home safe for them.

## INTRODUCTION

In the United States, the annual incidence of animal bites is 2 to 5 million, accounting for about 1% of all emergency departments visits and resulting in 20 to 35 deaths, primarily among infants and small children [1]. The vast majority of mammalian bites are inflicted by dogs (60%-90%). Herein we report a case of dog bite in a male child affecting external genitalia.

## CASE REPORT

A 3-month-old male was brought to the emergency room of another hospital after genital trauma caused by bite of miniature Dachshund. His mother was temporarily caring for the dog in the absence of his owner. Upon returning home, the patient’s father found that child had sustained a scrotal avulsion injury requiring immediate medical attention. However, the patient’s mother was so intoxicated that she was completely unaware of the situation. The patient presented in ER with pallor, tachycardia (218 beats/min), and hypotension (69/29 mmHg). Parenteral antibiotics (sulbactam/ampicillin) and tetanus immunoglobulin were given. Physical examination in OR revealed absence of the scrotal and penile skin, with partial amputation of the glans (Fig. 1). Neither testis was palpable in the scrotal or inguinal region. Even after thorough inspection, neither of the spermatic cord stumps could be identified. Urethral patency was assured after insertion of a Foley catheter. Primary suture of the wound was not performed because scrotal and penile skin loss was substantial.

The patient was transferred to our hospital for further management of the wound on postoperative day 8. Wound care included saline irrigation, application of trafermin spray, and petroleum gauze dressing. The defect of the genital skin gradually decreased in size with time. The Foley catheter was removed on postoperative day 30, and the patient was able to void spontaneously with a good stream. The levels of follicle stimulating hormone, luteinizing hormone, and anti-Mullerian hormone were 9.95 mIU/mL, 62.09 mIU/mL, and <0.1 ng/mL, respectively; these results confirmed bilateral testicular loss. The patient was discharged on postoperative day 38 to a protective institution, where he has been doing well. The penis was buried due to contracture of the genital skin 8 months after the trauma (Fig.1).

**Figure:**
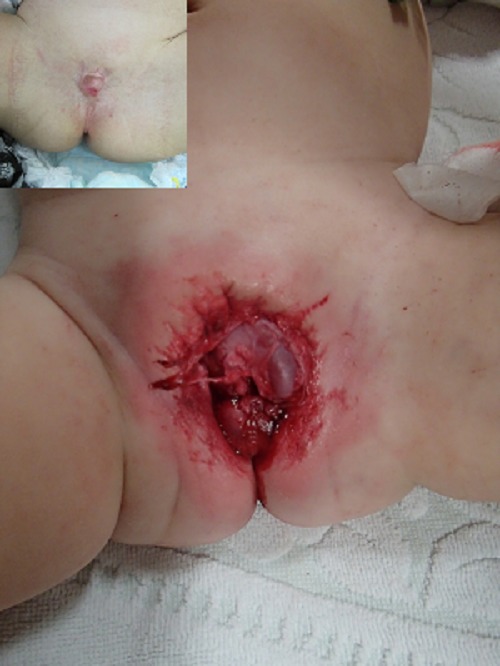
Figure 1: Absence of the scrotal and penile skin, with partial amputation of the glans; Inset shows penis was buried due to contracture of the genital skin 8 months after the trauma.

## DISCUSSION

Dog bites to the head and neck occur in 60%–70% of attacks in children below the age of 5 years.[2,7] On the other hand, genital trauma caused by dog bites is rare. Only 10 cases of scrotal dog bites in children are reported.[3-7] Five of these cases included infants, and all of them suffered unilateral or bilateral testicular loss like our patient. Small body size and the vulnerability of infants are believed to be associated with the severity of the genital trauma, including testicular loss. As an initial treatment, all patients underwent irrigation, debridement (if possible), and primary wound suturing. One patient underwent genital reconstruction in conjunction with the initial treatment. Another patient underwent female genitoplasty one year after the trauma based on psychological evaluation of the patient and family. This reassigned patient should be followed for a long time, considering the potential for gender identity issues. We have no intention of reassigning our patient a female sex although he suffered complete avulsion of the scrotum and bilateral testicular loss. It is because prenatal androgens are thought to influence the development of male sexual identity.[8,9] We are planning to perform genital reconstruction and hormone replacement in the future.

Safety of the child in home environment is the main concern. Many children are returned to dangerous home environments only to be repeatedly neglected or physically abused.[3] In conclusion, clinicians should be alert and attentive to the child’s home environment when managing children with genital trauma.

## Footnotes

**Source of Support:** Nil

**Conflict of Interest:** None declared

